# Gut virome dysbiosis impairs antitumor immunity and reduces 5-fluorouracil treatment efficacy for colorectal cancer

**DOI:** 10.3389/fonc.2024.1501981

**Published:** 2024-12-19

**Authors:** Hui Huang, Ying Yang, Xiaojiao Wang, Biao Wen, Xianglan Yang, Wei Zhong, Qiurong Wang, Feng He, Jun Li

**Affiliations:** ^1^ Department of Clinical Medicine, Chengdu Medical College, Chengdu, Sichuan, China; ^2^ Department of Gastroenterology, First Affiliated Hospital of Chengdu Medical College, Chengdu, Sichuan, China; ^3^ Department of Gastroenterology, Fifth People’s Hospital of Sichuan Province, Chengdu, Sichuan, China; ^4^ First Affiliated Hospital of Chengdu Medical College, Pengzhou Second People’s Hospital, Chengdu, China

**Keywords:** colorectal cancer, 5-fluorouracil, gut virome, fecal microbiota transplantation, immune response

## Abstract

**Introduction:**

Despite the established influence of gut bacteria, the role of the gut virome in modulating colorectal cancer (CRC) patient chemotherapy response remains poorly understood. In this study, we investigated the impact of antiviral (AV) drug-induced gut virome dysbiosis on the efficacy of 5-FU in CRC treatment.

**Methods:**

Using a subcutaneous CRC mouse model, we assessed tumor growth and immune responses following AV treatment, fecal microbiota transplantation (FMT), and 5-FU administration.

**Results:**

AV therapy reduced the abundance of gut DNA and RNA viruses, leading to accelerated tumor growth, shortened survival, and diminished chemotherapy efficacy. FMT restored the gut virome, improving tumor suppression and extending the survival of 5-FU-treated mice. Metagenomic sequencing revealed significant changes in virome composition, AV treatment expanded *Kahnovirus*, *Petivirales*, and *Enterogokushovirus*, whereas FMT enriched *Peduovirus STYP1*, *Mahlunavirus rarus*, and *Jouyvirus ev207*. AV treatment reduced the number of dendritic cells and CD8+ T cells in peripheral blood and tumor tissues, impairing antitumor immunity, FMT reversed these deficiencies. To further investigate the underlying mechanisms, we examined the TLR3-IRF3-IFN-β pathway, essential for recognizing viral RNA and triggering immune responses. AV treatment downregulated this pathway, impairing immune cell recruitment and reducing chemotherapy efficacy, while activation of TLR3 with Poly(I:C) restored pathway function and enhanced the effectiveness of 5-FU.

**Discussion:**

These findings suggest the importance of maintaining gut virome integrity or activating TLR3 as adjunct strategies to enhance chemotherapy outcomes in CRC patients.

## 1 Introduction

Colorectal cancer (CRC) is one of the most common malignant tumors of the digestive tract. According to statistics, over 1.9 million new cases of colorectal cancer arise worldwide each year and approximately 935,000 deaths occur, accounting for one-tenth of all cancer-related deaths (1). Surgical resection is curative for most early-stage patients, but chemotherapy remains the most common treatment modality for patients with advanced-stage colon cancer. The first-line clinical chemotherapy regimen is traditional 5-fluorouracil (5-FU)-based therapy. 5-FU is a cytotoxic drug that targets thymidylate synthase, thereby inducing double-stranded RNA and DNA breaks, causing cell cycle arrest and apoptosis and subsequently inhibiting cancer cell growth (2). However, the treatment efficacy of 5-FU in advanced colon cancer patients is only 10% to 15%, and recurrence rates of up to 30% in stage I-III patients and up to 65% in stage IV patients occur after chemotherapy (3, 4). Therefore, improving the efficacy of chemotherapy for treating CRC has become a critical clinical issue.

The human gut microbiome comprises bacteria, viruses, fungi, archaea, and parasites, and the total number of these cells usually exceeds that of the host cells (5). Homeostasis of the gut microbiome is considered beneficial for health; however, gut microbiota dysbiosis poses a threat to host health and can lead to various diseases, including inflammatory bowel disease, chronic liver disease, obesity, diabetes, hypertension, etc. (6). Studies have shown that gut bacteria are closely related to the occurrence and development of CRC and can modulate the antitumor response to chemotherapeutic drugs. In animal experiments, administration of a mixture of antibiotics, including vancomycin, imipenem, and neomycin, to deplete the gut microbiota impaired the anticancer effects of the cytotoxic drugs oxaliplatin and cisplatin in CT26 subcutaneous tumor-bearing mice (7). Furthermore, the use of antibiotics in patients with metastatic CRC before starting 5-FU-based chemotherapy is associated with shorter progression-free survival and overall survival (8).

In addition to gut bacteria, gut viruses are also important components of the gut microbiome. The human gut virome is primarily composed of bacteriophages, eukaryotic viruses, archaeal viruses, and endogenous retroviruses. Given that commensal viruses participate in host immune development and maturation, dysbiosis of these viruses may be closely associated with CRC (9). Studies have revealed that the gut viromes of CRC patients are altered compared with those of healthy subjects, with enrichment of several genera, including *Orthobunyavirus*, *Inovirus*, and *Tunalikevirus*, and that the composition of the gut virome is closely related to CRC stage and prognosis (10). In addition, multiple studies have shown that human papillomavirus (HPV), cytomegalovirus (CMV), and human polyomavirus 2 (JCV) may be associated with an increased incidence of CRC (11). Gogokhia et al. reported that a tail-shaped bacteriophage isolated from patients with active ulcerative colitis inhibited the growth of carcinogenic adherent-invasive *Escherichia coli* and suppressed intestinal tumor growth in a mouse model (12). To date, no consistent conclusion has been reached regarding the role of gut viruses in the occurrence and development of CRC. Moreover, studies regarding whether gut viruses affect the efficacy of chemotherapy for CRC treatment have not been reported. Therefore, in this study we aimed to investigate the impact of gut viruses in conjunction with 5-FU treatment on antitumor efficacy in patients with CRC.

Our study revealed that administration of an antiviral cocktail therapy including ribavirin (10 mg/kg), lamivudine (30 mg/kg), and acyclovir (20 mg/kg) induced gut virome dysbiosis, which promoted CRC growth in mice and reduced the antitumor effects of 5-FU. Furthermore, depletion of the gut virome significantly reduced the number of dendritic cells (DCs) and CD8+ T cells in peripheral blood and tumor tissues, whereas no significant difference was observed in the number of CD4+ T cells. The restoration of the gut microbiota through fecal microbiota transplantation (FMT) inhibited the progression of CRC and reversed the weakened antitumor effect of 5-FU. Moreover, the numbers of DCs and CD8+ T cells in the peripheral blood and tumor tissues were significantly increased. To further investigate the mechanistic underpinnings of gut virome involvement, we analyzed the TLR3-IRF3-IFN-β signaling pathway, which plays a crucial role in innate immunity by recognizing viral RNA and initiating an immune response. We hypothesized that antiviral treatment would downregulate this pathway, thus impairing immune cell recruitment and weakening the efficacy of 5-FU chemotherapy. In addition, we explored whether the TLR3 agonist Poly(I:C) could restore this pathway’s activity and enhance the therapeutic effects of 5-FU. This work helps to fill the research gap regarding the effects of gut viruses on the efficacy of chemotherapy for CRC treatment and further enhances our understanding of the relationship between the gut microbiome and tumor treatment, thereby improving the efficacy of antitumor drugs to benefit CRC patients.

## 2 Materials and methods

### 2.1 Subjects and sample collection

This study was approved by the Ethics Committee of the First Affiliated Hospital of Chengdu Medical College (2024CYFYIRB-BA-Jul17), and informed consent was obtained from all participants prior to their inclusion in the study. A total of 10 CRC patients meeting the inclusion criteria were recruited between January 2023 and June 2024, including 4 males and 6 females, aged 45 to 64. Fresh fecal samples were collected before and after 2 cycles of chemotherapy (The chemotherapy regimen consists of oxaliplatin combined with leucovorin and fluorouracil), stored at -80°C, and subsequently analyzed via metagenomic sequencing. Tumor size was assessed by imaging (CT or MRI) before and after chemotherapy according to RECIST 1.1 criteria, and its correlation with changes in the gut virome was analyzed.

### 2.2 Drugs and cell lines

5-FU, ribavirin, lamivudine, and acyclovir were purchased from MedChemExpress (USA). MC38 mouse colon cancer cells were obtained from West China Hospital, Sichuan University (China).

### 2.3 Experimental design and grouping

Throughout the experiment, internationally recognized animal experimentation guidelines were followed, and all animal experiments were approved by the Animal Experiment Ethics Committee of Chengdu Medical College (2022CYA-003). A total of 40 female C57BL/6 mice, aged 6-8 weeks and weighing 19.7 ± 2 g, were purchased from Chengdu Dashuo Experimental Animal Co., Ltd. The mice were housed under specific pathogen-free (SPF) conditions with a 12-h light/dark cycle and were allowed free access to food and water. The mice were divided into different treatment groups: 1) Antiviral group (AV): received oral antiviral cocktail therapy including ribavirin (10 mg/kg), lamivudine (30 mg/kg), and acyclovir (20 mg/kg) for 10 consecutive days (13); 2) Nonantiviral group (V): received oral PBS for 10 consecutive days; 3) Antiviral + 5-FU group (AV+FU): received antiviral cocktail therapy combined with intraperitoneal injection of 5-FU (25 mg/kg) for 5 consecutive days; 4) Nonantiviral + 5-FU group (V+FU): received oral PBS combined with intraperitoneal injection of 5-FU (25 mg/kg); 5) Antiviral + FMT group (AV+FMT): received antiviral cocktail therapy combined with 200 μl of fresh fecal suspension orally; and 6) Antiviral + PBS group (AV+PBS): received antiviral cocktail therapy combined with 200 μl of PBS orally; 7) Antiviral + FMT + 5-FU group (AV+FMT+FU): received antiviral cocktail therapy, 200 μl of fresh fecal suspension orally, and intraperitoneal injection of 5-FU (25 mg/kg); 8) Antiviral + PBS + 5-FU group (AV+PBS+FU): received antiviral cocktail therapy, 200 μl of PBS orally, and intraperitoneal injection of 5-FU (25 mg/kg); 9) Poly group: received antiviral cocktail therapy, followed by intraperitoneal injections of 5-FU (25 mg/kg for 5 consecutive days) and the TLR3 agonist Poly(I:C) [10 μg/mouse, administered twice every 5 days until the endpoint (14)] after tumor appearance; and 10) no-Poly group: received antiviral cocktail therapy, followed by intraperitoneal injections of 5-FU (25 mg/kg for 5 consecutive days) and an equivalent volume of PBS (administered twice every 5 days until the endpoint) after tumor appearance. MC38 colon cancer cells were cultured in a humidified atmosphere of 5% CO2 at 37°C. To establish a mouse model of subcutaneous CRC tumors, tumor cells were diluted to 5×10^6^ cells/ml and injected subcutaneously into the right axillae of C57BL/6 mice. Tumor size was monitored every 3 days, and the tumor volume (mm^3^) was calculated as follows: (longest diameter) × (shortest diameter)^2^/2. Tumor growth curves and survival curves were plotted. At the study endpoint, the mice were anesthetized with isoflurane via a gas anesthesia machine. Fecal, peripheral blood, and tumor tissue samples were collected, and the mice were euthanized.

### 2.4 Fecal microbiota transplantation

Fresh feces were collected from healthy donor mice. Approximately 1 g feces was placed in a 15 ml centrifuge tube, and 5 ml sterile 0.9% NaCl solution (37°C) was added. The mixture was homogenized, and the homogenate was centrifuged at 800 × g for 2 min. The supernatant was collected for FMT. FMT was performed every other day, with each mouse receiving 200 μl of fresh fecal suspension orally for 2 weeks.

### 2.5 VLP staining of fecal samples

Fresh fecal samples were collected from the mice after antiviral cocktail therapy or FMT treatment. A 0.5 g fecal sample was suspended in 10 ml 0.02 μm-filtered sterile magnesium salt buffer solution. The suspension was centrifuged at 2500 rpm for 10 min at 4°C, and the supernatant was filtered through 0.45 μm and 0.22 μm syringe filters to remove the cells. The filtrate was then diluted 10-fold and filtered through a 0.02 μm filter. The filter was stained with 10X SYBR Gold (Thermo, USA) and 10X SYBR Green II (Thermo, USA) for 15 min. After washing, the fluorescence was visualized under a microscope, and images were captured and quantified.

### 2.6 Metagenomic sequencing of fecal samples

Genomic DNA was extracted from the fecal samples using a HiPure Fecal DNA Kit (Magen, catalog number D3141) according to the manufacturer’s instructions. The steps were as follows: (1) extraction and quality control of the fecal microbial metagenome, (2) random fragmentation of DNA, (3) construction of a standard DNA-seq library, (4) library quality control and quantification, and (5) HiSeq platform sequencing to obtain FastQ data.

### 2.7 Flow cytometry analysis of peripheral blood DC cells

Five microliters of anti-mouse CD11c antibody (Elabscience, catalog number AF10866) and 5 μL of anti-mouse MHC II antibody (Elabscience, catalog number AF11357) were added to 100 μL of noncoagulated mouse blood in a flow cytometry tube, mixed, and incubated on ice for 30 min. Red blood cells were lysed with 2 ml lysis buffer on ice for 10 min, and the suspension was subsequently centrifuged at 300 × g for 5 min. The supernatant was discarded, and the cells were washed with 2 ml PBS and centrifuged at 300 × g for 5 min. The cells were resuspended in 500 μl 1× flow cytometry staining buffer and analyzed by flow cytometry.

### 2.8 Flow cytometry analysis of peripheral blood CD4+ and CD8+ T cells

One hundred microliters of single-cell suspension was added to a flow cytometry tube, along with 5 μl anti-mouse CD4 antibody (Elabscience, catalog number AF15231) and 5 μl anti-mouse CD8a antibody (Elabscience, catalog number AF13226). The mixture was vortexed, incubated on ice for 30–60 min, and lysed with 2 ml 1× red blood cell lysis buffer. The cells were subsequently centrifuged at 300 × g for 5 min, washed with 2 ml PBS, and resuspended in 500 μl 1× flow cytometry staining buffer for flow cytometry analysis.

### 2.9 Immunohistochemistry

To assess immune cell infiltration in tumor tissues, IHC was performed to detect CD4, CD8, and CD11c expression. Frozen slides were placed in a 37°C oven for 10-20 min, fixed in paraformaldehyde for 30 min, and subjected to antigen retrieval in EDTA antigen retrieval buffer. The slides were blocked with 3% BSA, incubated overnight at 4°C with primary antibodies against CD4 (Cell Signaling Technology, catalog number 25229S), CD8α (Cell Signaling Technology, catalog number 98941S), and CD11c (Cell Signaling Technology, catalog number 97585), and then labeled with horseradish peroxidase (HRP) for visualization. Staining scores were determined based on the intensity and percentage of positively stained cells. The percentage of positive cells was categorized into four levels: 0 (<5% positive), 1 (<25% positive), 2 (25–50% positive), 3 (51–75% positive), and 4 (>75% positive). Staining intensity was rated as follows: 0 (no staining), 1 (weak staining), 2 (moderate staining), and 3 (strong staining). To ensure accurate representation, 10 fields per slide were randomly selected and examined at ×100 magnification, with an average score calculated to provide an overall expression level (15).

### 2.10 Western blot analysis

To evaluate the expression levels of TLR3, IRF3, p-IRF3, and IFN-β proteins, we performed Western blotting on tumor tissue samples. Protein was extracted using RIPA lysis buffer (Thermo Fisher Scientific, Cat# 89900), and protein concentration was determined with the BCA Protein Assay Kit (Beyotime, Cat# P0012). Equal amounts of protein were separated by SDS-PAGE and transferred onto a PVDF membrane (Millipore, Cat# IPVH00010). The membranes were blocked with 5% non-fat milk for 1 hour at room temperature. Primary antibodies used were TLR3 (Abcam, Cat# ab62566), IRF3 (Cell Signaling Technology, Cat# 4302), p-IRF3 (Cell Signaling Technology, Cat# 4947), and IFN-β (Thermo Fisher Scientific, Cat# PA5-20390), with β-Actin (Abcam, Cat# ab8226) as the loading control. Membranes were incubated with primary antibodies overnight at 4°C, followed by incubation with HRP-conjugated secondary antibodies (Abcam, Cat# ab97051) for 1 hour at room temperature. Bands were visualized using an ECL detection kit (Thermo Fisher Scientific, Cat# 32106), and band intensities were analyzed with ImageJ software.

### 2.11 Immunofluorescence analysis

Immunofluorescence staining was performed to assess the localization and expression of TLR3, p-IRF3, and IFN-β in tumor tissues. Tumor samples were fixed in 4% paraformaldehyde and embedded in paraffin. Following deparaffinization and rehydration, the sections were permeabilized with 0.3% Triton X-100 for 10 minutes, then blocked with 10% goat serum (Sigma-Aldrich, Cat# G9023) for 1 hour at room temperature. The sections were incubated overnight with primary antibodies for TLR3 (Abcam, Cat# ab62566), p-IRF3 (Thermo Fisher Scientific, Cat# PA5-105648), and IFN-β (Thermo Fisher Scientific, Cat# PA5-20390). The next day, sections were incubated with Alexa Fluor 488- or Alexa Fluor 594-conjugated secondary antibodies (Thermo Fisher Scientific, Cat# A-11001 for Alexa Fluor 488 and Cat# A-11012 for Alexa Fluor 594) for 1 hour at room temperature. DAPI (Beyotime, Cat# C1002) was used for nuclear staining. Images were captured with a fluorescence microscope, and fluorescence intensity was quantified using ImageJ software.

### 2.12 Statistical analysis

Statistical analysis was performed via GraphPad Prism 8.0.2 software. Quantitative data with a normal distribution are expressed as the means ± standard deviations (x ± s), and comparisons among three groups were performed via one-way ANOVA. Nonnormally distributed data were analyzed via the Kruskal-Wallis test. Chi-square tests were used to compare qualitative data between groups. Statistical significance was defined as P<0.05.

## 3 Results

### 3.1 Associations between the gut virome and chemotherapy efficacy in CRC patients

**Figure 1:**
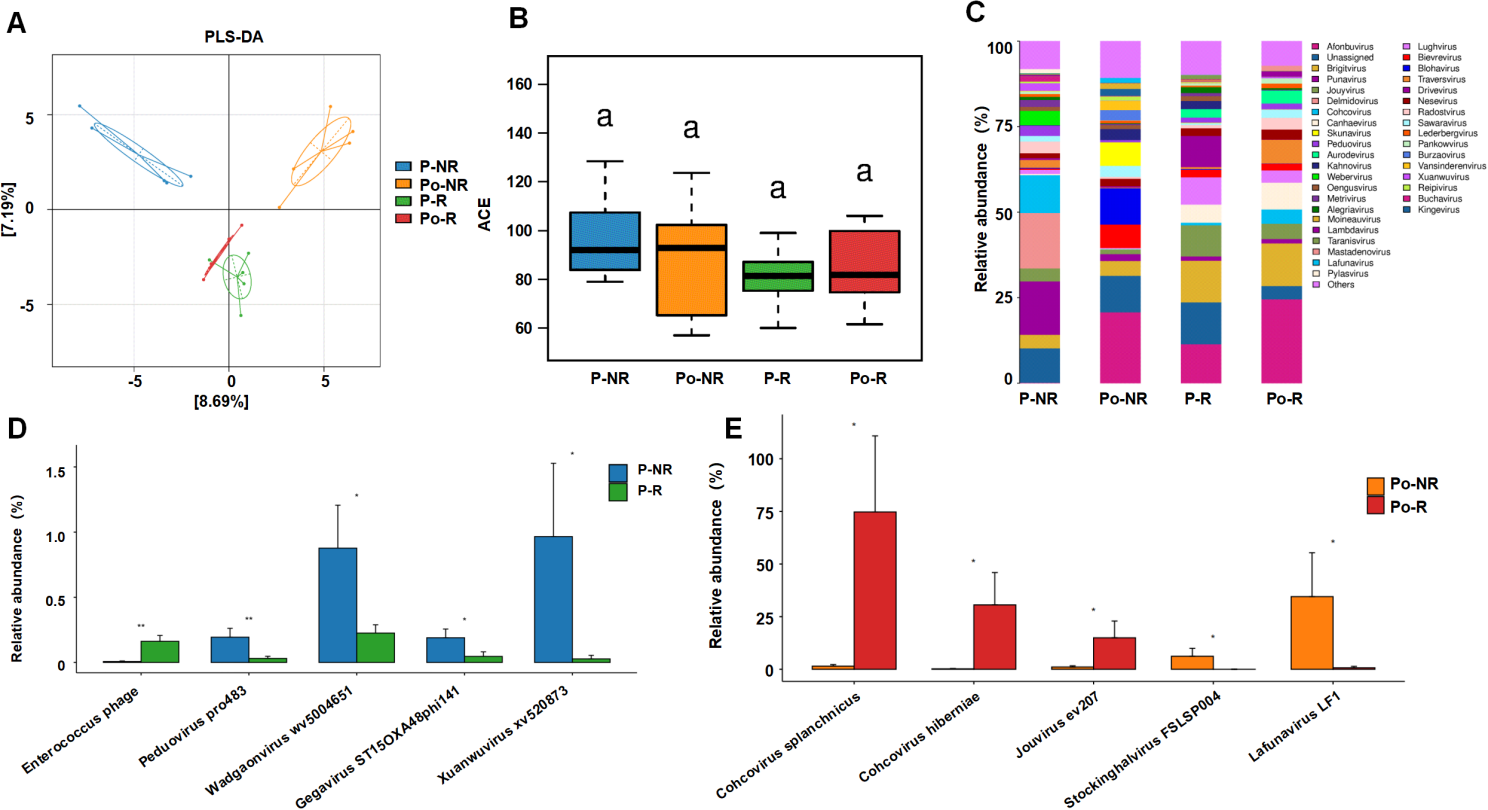
Associations between the gut virome and chemotherapy efficacy in CRC patients. **(A)** PLS-DA plot showing the clustering of viral communities in different sample groups (P-NR, Po-NR, P-R, and Po-R). **(B)** Box plot of ACE indices representing α diversity across different sample groups. Statistical significance is indicated by letters (a). **(C)** Stacked bar charts showing the relative abundance of various viral taxa in different sample groups. **(D)** Bar graphs comparing the relative abundance of specific viral species between the P-NR and P-R groups. **(E)** Bar graphs comparing the relative abundance of specific viral species between the Po-NR and Po-R groups. *, P < 0.05; **, P < 0.01.

To investigate the association between the gut virome and chemotherapy efficacy in CRC patients, we collected fecal samples from 10 CRC patients before and after chemotherapy. The baseline characteristics of the patients are shown in **Table 1**. According to the RECIST 1.1 criteria for tumor assessment, these patients were divided into responders (stable disease in 4 patients, partial response in 1 patient) and nonresponders (disease progression in 5 patients). The sample groups were as follows: prechemotherapy nonresponders (P-NR), prechemotherapy responders (P-R), postchemotherapy nonresponders (Po-NR), and postchemotherapy responders (Po-R). The PLS-DA plot was generated based on the relative abundances of viral genera identified from fecal metagenomic sequencing data. The viral abundance matrix was normalized, log-transformed, and used as input for PLS-DA analysis to visualize the differences in gut virome composition among the sample groups. **Figure 1A** shows distinct clustering of viral communities among the sample groups. The P-R and P-NR groups clustered separately from the Po-R and Po-NR groups, indicating significant differences in viral composition before and after chemotherapy. Additionally, the viral communities in the P-R and Po-R groups were similar, indicating that the viral communities of the responders were relatively stable. The ACE index (**Figure 1B**) shows the α diversity across different sample groups. We found that the viral diversity of nonresponders before and after chemotherapy was greater than that of responders, although the difference was not statistically significant. Additionally, there was no significant difference in viral diversity among CRC patients before and after chemotherapy, suggesting that chemotherapy has no significant impact on gut viral diversity.

**Table 1:** Basic information of colorectal cancer patients.

Patient ID	Gender	Age	Cancer Stage	Chemotherapy Regimen	Comorbidities	ECOG Score
1	Male	55	III	FOLFOX (5-FU + Oxaliplatin)	Hypertension, Diabetes	1
2	Female	53	II	FOLFIRI (5-FU + Irinotecan)	None	0
3	Male	48	III	FOLFOX (5-FU + Oxaliplatin)	Coronary artery disease, Stroke	2
4	Male	60	III	FOLFOX (5-FU + Oxaliplatin)	Hyperlipidemia	1
5	Female	56	II	FOLFIRI (5-FU + Irinotecan)	None	0
6	Female	52	III	FOLFIRI (5-FU + Irinotecan) + Bevacizumab	Hyperthyroidism, Asthma	1
7	Male	64	IV	FOLFIRI (5-FU + Irinotecan) + Cetuximab	Hypertension, Chronic kidney disease	2
8	Female	58	II	FOLFOX (5-FU + Oxaliplatin)	Asthma, Osteoporosis	0
9	Female	45	III	FOLFOX (5-FU + Oxaliplatin)	None	0
10	Female	62	IV	FOLFOX (5-FU + Oxaliplatin) + Bevacizumab	Osteoporosis, Diabetes	2

ECOG, Eastern Cooperative Oncology Group performance status score was used to evaluate each patient’s performance status at enrollment, ranging from 0 to 5. A score of 0 indicates the patient is fully active and can carry out all normal activities without restriction. 1 means the patient is ambulatory but restricted in strenuous activity, able to perform light work. 2 signifies the patient can perform all self-care tasks but is unable to engage in work activities and is up for more than 50% of their waking hours. 3 indicates the patient has limited self-care ability and is confined to a bed or chair for more than 50% of the day. 4 represents a completely disabled patient who cannot perform any self-care and is totally confined to a bed or chair. Finally, 5 represents death.

**Figure 1C** illustrates the relative abundance of various viral taxa in different sample groups. There were notable differences in the viral taxon composition among the groups. The viral community in the P-NR group was highly diverse and was composed of genera such as *Punavirus*, *Delmidovirus*, and *Cohcovirus*. In the Po-NR group, the relative abundances of *Afonbuvirus* and *Brigitvirus* increased, whereas the abundance of *Punavirus* decreased, suggesting that chemotherapy may have promoted the expansion of certain viral genera. Compared with those in the P-NR group, the viral genera in the P-R group were dominated by *Afonbuvirus* and *Brigitvirus*, with lower abundances of *Punavirus* and *Delmidovirus*. In the Po-R group, the abundance of *Brightvirus* and *Ardovirus* increased, but the overall viral community structure changed little, indicating that the abundances of these viral genera remained relatively stable after chemotherapy. Further analysis of the significantly differentially abundant viral taxa before and after chemotherapy in nonresponders and responders revealed that, before chemotherapy, *Enterococcus phage* was significantly enriched in the P-R group, whereas *Peduovirus pro483*, *Wadgaonvirus wv5004651*, *Gegavirus ST15OXA48phi141*, and *Xuanwuvirus xv520873* were significantly enriched in the P-NR group (**Figure 1D**). After chemotherapy, *Cohcovirus splanchnicus*, *Cohcovirus hiberniae*, and *Jouyvirus ev207* were significantly enriched in the Po-R group, whereas *Stockinghalvirus FSLSP004* and *Lafunavirus LF1* were significantly enriched in the Po-NR group (**Figure 1E**). These findings suggest that certain viral taxa may be associated with differences in chemotherapy outcomes in patients with CRC. Observed variations in viral enrichment between responders and nonresponders, both before and after treatment, could offer preliminary insights into potential biomarkers for predicting chemotherapy responses. However, further investigation is needed to establish a direct relationship between these viral taxa and chemotherapy effectiveness.

### 3.2 Antiviral treatment impairs the efficacy of 5-FU treatment against colorectal tumors

**Figure 2:**
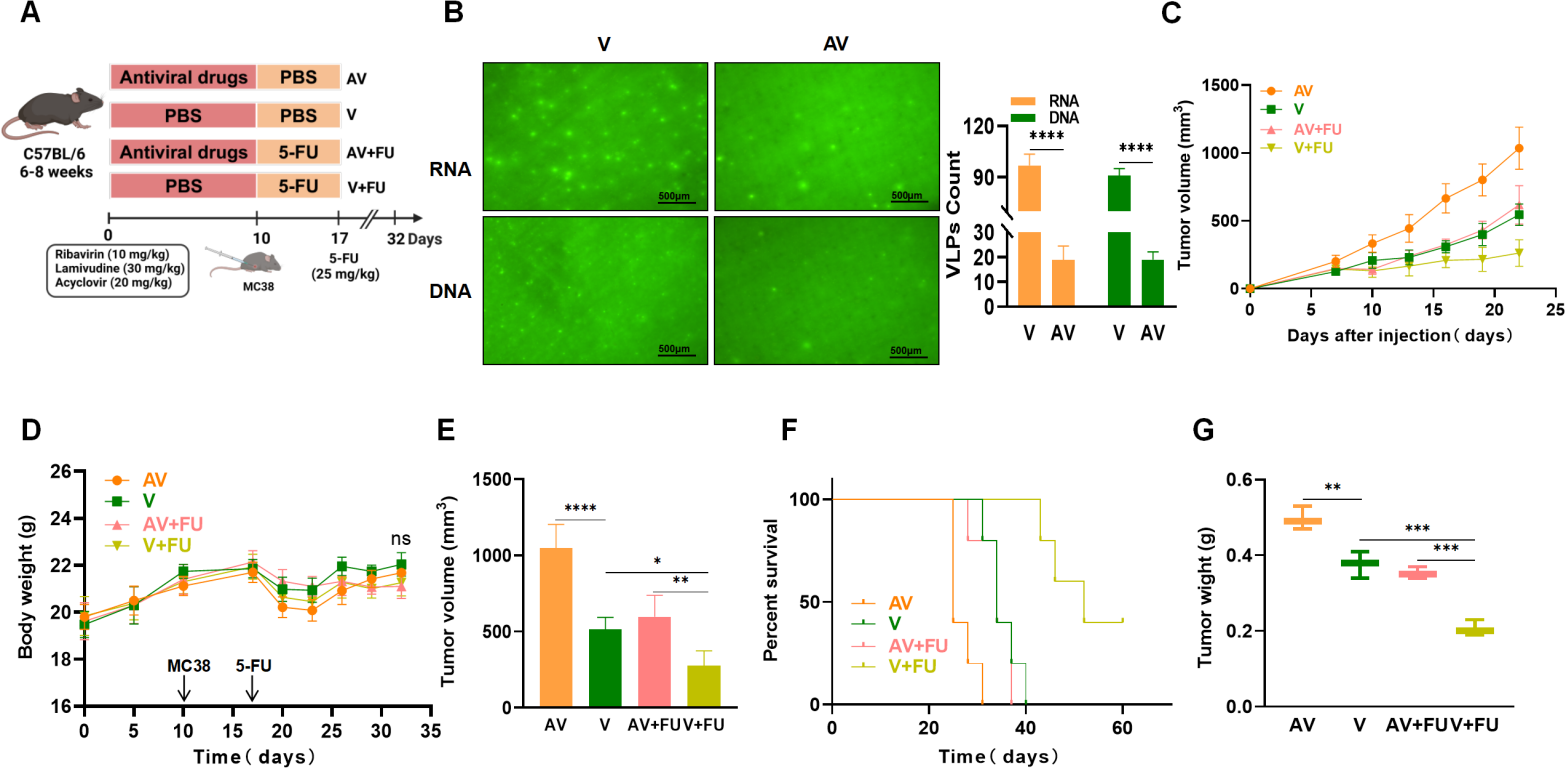
Antiviral (AV) treatment impairs the efficacy of 5-FU treatment against colorectal tumors. **(A)** Experimental design. **(B)** Fluorogram of DNA and RNA virus-like particle staining of mouse feces before and after AV treatment and quantification. Scale bar=500 µm. **(C)** Tumor volume progression over time in different treatment groups. **(D)** Changes in body weight over time in different treatment groups. **(E)** Comparison of final tumor volumes after 22 days among the different groups. **(F)** Survival rates of the mice in each treatment group over time. **(G)** Comparison of tumor weights among the groups. n=5. *, P < 0.05; **, P < 0.01; ***, P < 0.001; ****, P < 0.0001.

To further investigate the impact of gut viruses on 5-FU treatment for CRC, we employed an antiviral cocktail therapy (ribavirin, lamivudine, and acyclovir) to eliminate gut viruses in mice. After establishing a subcutaneous xenograft model using MC38 mouse colon cancer cells, we treated the mice with 5-FU and observed tumor growth (**Figure 2A**). SYBR Gold staining (for DNA viruses) and SYBR Green II staining (for RNA viruses) were used to detect virus-like particles in feces to assess gut viral abundance. We observed a significant reduction in numbers of both DNA and RNA viruses after antiviral treatment compared with before treatment (**Figure 2B**). By comparing the tumor volumes and survival times between the antiviral group and the nonantiviral group, we found that by day 22, the tumor volumes in the antiviral group (AV) were significantly greater than those in the nonantiviral group (V) (**Figure 2C**), and the survival time in the antiviral group was significantly shorter than that in the nonantiviral group (**Figure 2F**). There were no significant differences in body weight changes among the groups throughout the experimental period (**Figure 2D**). These results indicate that antiviral treatment promotes colorectal tumor growth and decreases survival time in mice. Additionally, we assessed the effect of antiviral treatment on the chemotherapeutic efficacy of 5-FU treatment by comparing tumor size and survival time between mice in the AV+FU and V+FU groups. The tumor volume in the V+FU group was significantly smaller than that in the nonantiviral group by day 22 (**Figures 2C, E**), and the survival time was extended (**Figure 2F**), suggesting that 5-FU effectively inhibited colorectal tumor growth. However, the therapeutic effect of 5-FU was reduced following antiviral treatment. The tumor volume in the AV+FU group was significantly greater than that in the V+FU group by day 22 (**Figure 2E**), and the survival time was shorter (**Figure 2F**), indicating that antiviral treatment diminishes the inhibitory effect of 5-FU on colorectal tumor growth. Furthermore, the tumor weight in the V+FU group was significantly lower than that in the AV+FU group (**Figure 2G**). These findings further support that antiviral treatment promotes colorectal tumor growth in mice and diminishes the efficacy of 5-FU treatment.

### 3.3 FMT enhances the efficacy of 5-FU treatment against colorectal tumors

**Figure 3:**
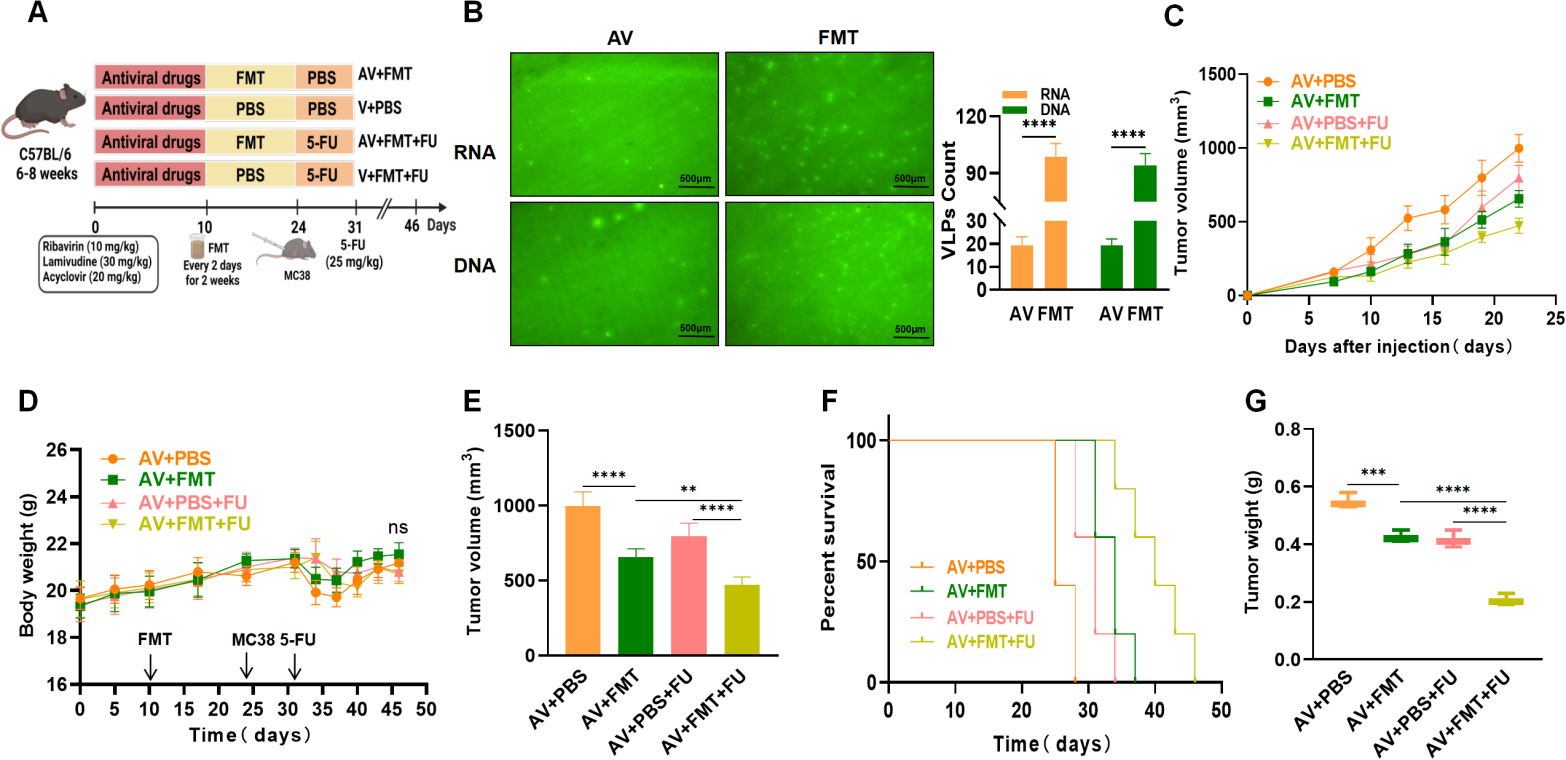
Fecal microbiota transplantation (FMT) enhances the efficacy of 5-FU treatment against colorectal tumors. **(A)** Experimental design. **(B)** Fluorogram of DNA and RNA virus-like particle staining of mouse feces before and after FMT and quantification. Scale bar=500 µm. **(C)** Tumor volume progression over time in different treatment groups. **(D)** Changes in body weight over time in different treatment groups. **(E)** Comparison of final tumor volume after 22 days among the different groups. **(F)** Survival rates of the mice in each treatment group over time. **(G)** Comparison of tumor weights among the groups. n=5. **, P < 0.01; ***, P < 0.001; ****, P < 0.0001.

To verify whether AV treatment affects the efficacy of 5-FU by modulating the gut microbiota, all the mice were subjected to AV drug treatment to eliminate gut viruses. The mice in the AV + FMT + 5-FU group received FMT followed by that with 5-FU, whereas the mice in the AV + PBS + 5-FU group received PBS treatment followed by that with 5-FU (**Figure 3A**). We observed a significant increase in numbers of both intestinal DNA and RNA viruses following FMT compared with those in the AV group (**Figure 3B**). We compared the tumor volumes and survival times among the various treatment groups. The tumor volumes in the AV+PBS group mice were significantly greater than those in the AV+FMT group mice by day 22 (**Figures 3C, E**), suggesting that FMT can inhibit tumor growth. No significant differences in body weight changes were observed among the groups throughout the experimental period (**Figure 3D**). When 5-FU was administered, the tumor volumes in the AV+PBS+5-FU group mice were significantly greater than those in the AV+FMT+5-FU group mice (**Figure 3E**), indicating that FMT enhances the efficacy of 5-FU treatment. Survival analysis revealed that mice in the AV+FMT+5-FU group had longer survival times than those in the AV+PBS+5-FU group did (**Figure 3F**). Furthermore, the tumor weights in the AV+PBS group mice were significantly greater than those in the AV+FMT group mice, and the tumor weights in the AV+PBS+5-FU group mice were significantly greater than those in the AV+FMT+5-FU group mice (**Figure 3G**). These findings indicate that FMT enhances the inhibitory effect of 5-FU treatment on colorectal tumor growth in mice and extends survival time, suggesting that modulation of the gut microbiota can improve the efficacy of chemotherapy.

### 3.4 Impact of AV drugs on the gut microbiota

**Figure 4:**
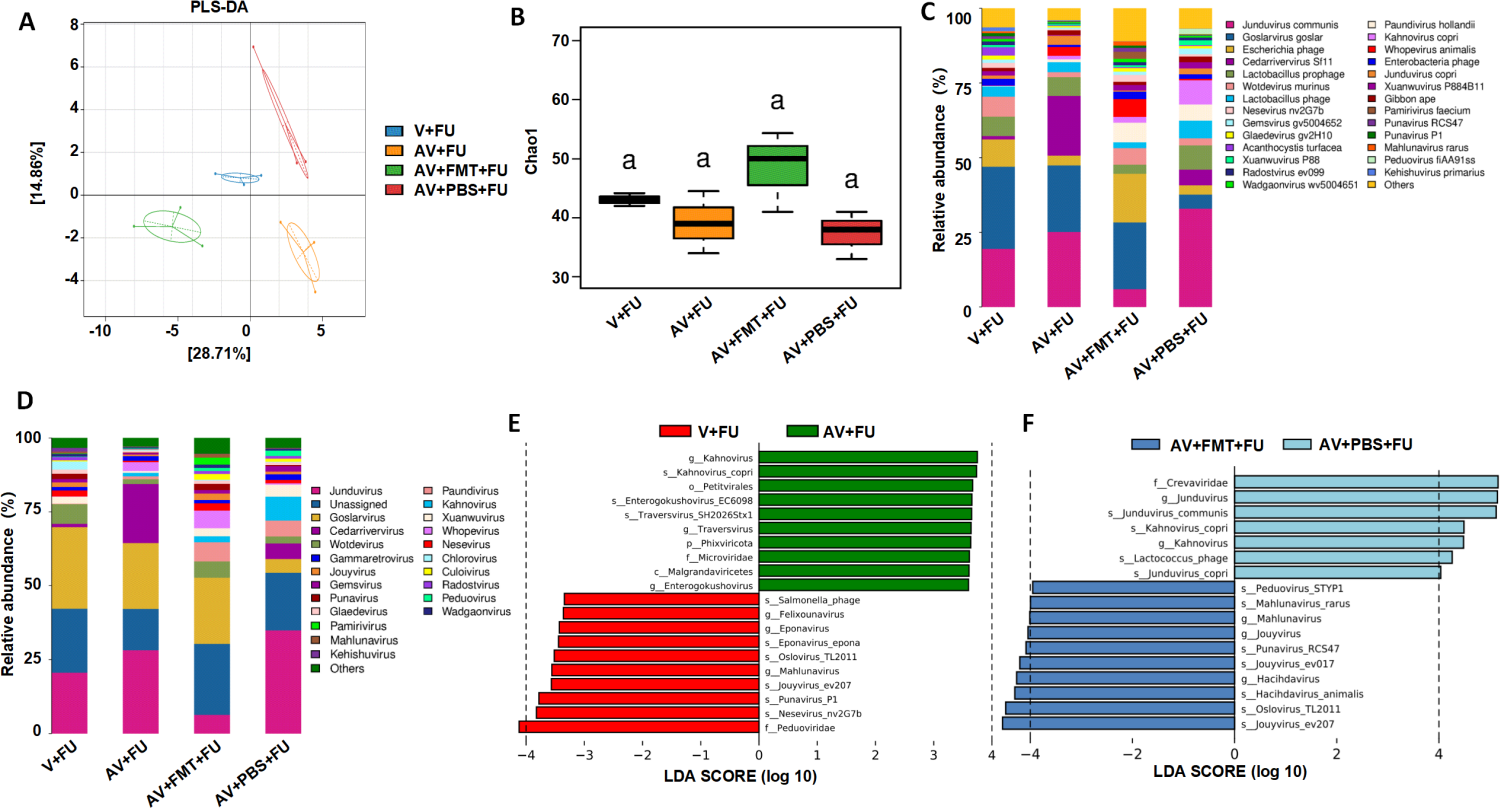
Impact of AV drugs on the gut virome. **(A)** PLS-DA plot showing the clustering of different treatment groups on the basis of viral community composition. **(B)** Chao1 index of gut viruses in different treatment groups. Statistical significance is indicated by letters (a). **(C)** Relative abundance of viral taxa across different treatment groups. **(D)** Relative abundance of specific viral families in each treatment group. **(E)** LEfSe analysis showing significantly differentially abundant viral taxa between the V+FU and AV+FU groups. **(F)** LEfSe analysis showing significantly differentially abundant viral taxa between the AV+FMT+FU and AV+PBS+FU groups.

To study the impact of AV drugs and FMT on the gut virome, we conducted metagenomic sequencing analysis on fecal samples from mice after gut virus depletion or FMT. The PLS-DA plot (**Figure 4A**) shows distinct clustering of viral communities among the V+FU, AV+FU, AV+FMT+FU, and AV+PBS+FU groups, indicating significant differences in gut virome composition. α diversity analysis via the Chao1 index (**Figure 4B**) revealed that the AV+FMT+FU group and the V+FU group presented higher median Chao1 indices than the AV+FU group and AV+PBS+FU group did, indicating greater viral richness in both groups, although the differences were not statistically significant. The analysis of intestinal viruses at the genus level (**Figure 4C**) revealed that the genera with the highest relative abundance across all groups were *Jundivirus communis*, *Goslarvirus goslar*, *Escherichia phage*, and *Cedarvirus Sf11*. The viral genus structures in the V+FU group and the AV+FMT+FU group were similar. Compared with those in the V+FU group and the AV+FMT+FU group, the relative abundances of *Jundivirus communis* and *Cedarvirus Sf11* increased in the AV+FU group and the AV+PBS+FU group, whereas the relative abundances of *Goslarvirus goslar* and *Escherichia phage* decreased. **Figure 4D** shows the changes in the relative abundances of specific viral species across the different groups. *Jundivirus*, *Goslarvirus*, and *Cedarvirus* included the species with the highest relative abundances in all the groups, collectively accounting for more than 50% of the total. The relative abundances of these genera varied under different treatment conditions. Compared with those in the V+FU group and the AV+FMT+FU group, the relative abundance of *Jundivirus* increased in the AV+FU and AV+PBS+FU groups, whereas the relative abundance of *Goslarvirus* decreased. Fecal microbiota transplantation (AV+FMT+FU) resulted in a species structure similar to that of the V+FU group.

LEfSe analysis (**Figure 4E**) revealed significant differences in viral taxa between the V+FU and AV+FU groups. In the V+FU group, taxa such as *Salmonella phage*, *Felixounavirus*, and *Eponavirus* were enriched, indicating that these viruses dominated the environment prior to AV treatment. In the AV+FU group, viral taxa such as *Kahnovirus*, *Petivirales*, *Enterogokushovirus*, *Traversvirus*, and *Phixviricota* were significantly enriched, reflecting the promoting effect of AV treatment on these viruses. Similarly, LEfSe analysis (**Figure 4F**) revealed significant differences between the AV+FMT+FU and AV+PBS+FU groups. In the AV+PBS+FU group, viral taxa such as the family *Crevaviridae*, the genera *Jundivirus* and *Kahnovirus*, and *Lactococcus phage* were significantly enriched. The combination of AV treatment and PBS may have promoted the expansion of these viruses, rendering them dominant in this group. In the AV+FMT+FU group, taxa such as *Peduovirus STYP1*, *Mahlunavirus rarus*, *Mahlunavirus*, *Jouyvirus*, *Punavirus RCS47*, *Hachidavirus*, *Oslovirus TL2011*, and *Jouyvirus ev207* were significantly enriched. These findings indicate that these viruses had high relative abundances in the environment following FMT, potentially rendering them dominant in this group. These findings suggest that FMT can modulate the gut virome composition and potentially restore a more beneficial viral community that enhances the efficacy of 5-FU chemotherapy in treating CRC.

**Figure 5:**
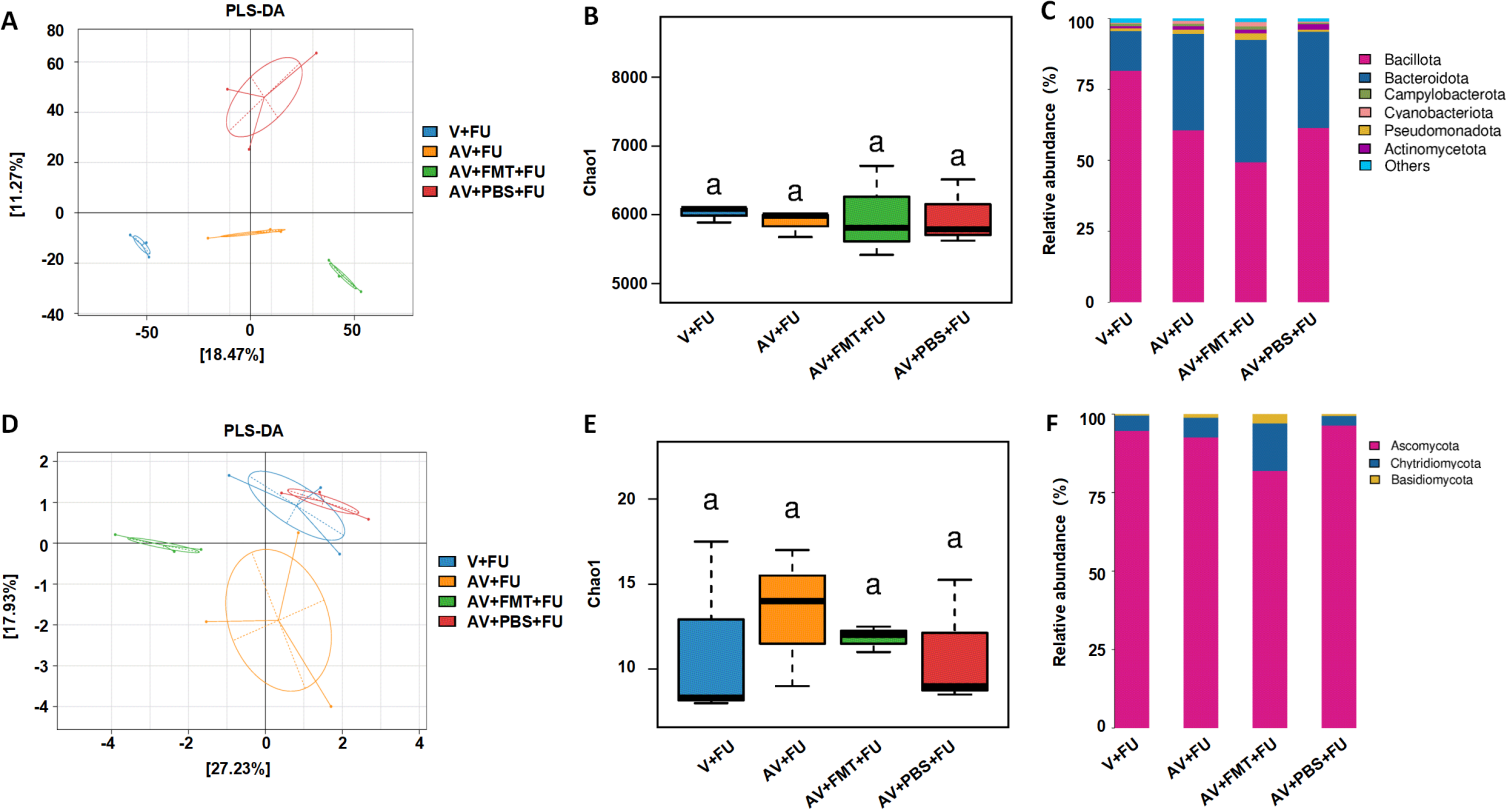
Impact of AV drugs on gut bacteria and the mycobiome. **(A)** PLS-DA plot showing the clustering of different treatment groups on the basis of bacterial community composition. **(B)** Chao1 indices of gut bacteria in different treatment groups. **(C)** Relative abundance of bacterial phyla across different treatment groups. **(D)** PLS-DA plot showing the clustering of different treatment groups on the basis of fungal community composition. **(E)** Chao1 indices of gut fungi in different treatment groups. **(F)** Relative abundance of fungal phyla across different treatment groups.

Through metagenomic analysis of the gut bacterial and fungal communities, we found that the bacterial and fungal communities in the V+FU, AV+FU, AV+FMT+FU, and AV+PBS+FU groups clustered distinctly, indicating significant differences in their composition (**Figures 5A, D**). α diversity analysis using the Chao1 index (**Figures 5B, E**) revealed no significant differences in bacterial or fungal diversity across the groups. The relative abundances of bacterial and fungal phyla (**Figures 5C, F**) highlighted changes in composition across the treatment groups. In the V+FU group, the gut microbiota was predominantly composed of *Bacillota* and *Bacteroidota*, with lower proportions of other phyla, such as *Cyanobacteria* and *Actinomycetota*. The AV+FU group exhibited an increased abundance of *Bacteroidota* and *Cyanobacteria*, indicating that AV treatment significantly altered the gut bacterial composition. In contrast, the AV+FMT+FU group displayed a more distinct microbial profile, with greater differences in bacterial communities compared to both the AV+FU and V+FU groups. This group showed higher proportions of *Bacteroidota* and *Pseudomonadota*, suggesting that FMT had a significant impact on these bacterial populations. Regarding fungal communities, the microbiota in the V+FU, AV+FU, AV+FMT+FU, and AV+PBS+FU groups was predominantly composed of *Ascomycota*, with minor contributions from *Basidiomycota* and *Chytridiomycota*. The AV+FMT+FU group showed a higher proportion of *Basidiomycota* and *Chytridiomycota* compared to the AV+PBS+FU group, suggesting that FMT supports the growth of these fungal communities.

### 3.5 The dysbiosis of the gut microbiota induced by AV drugs reduces DCs and CD8+ T cells

**Figure 6:**
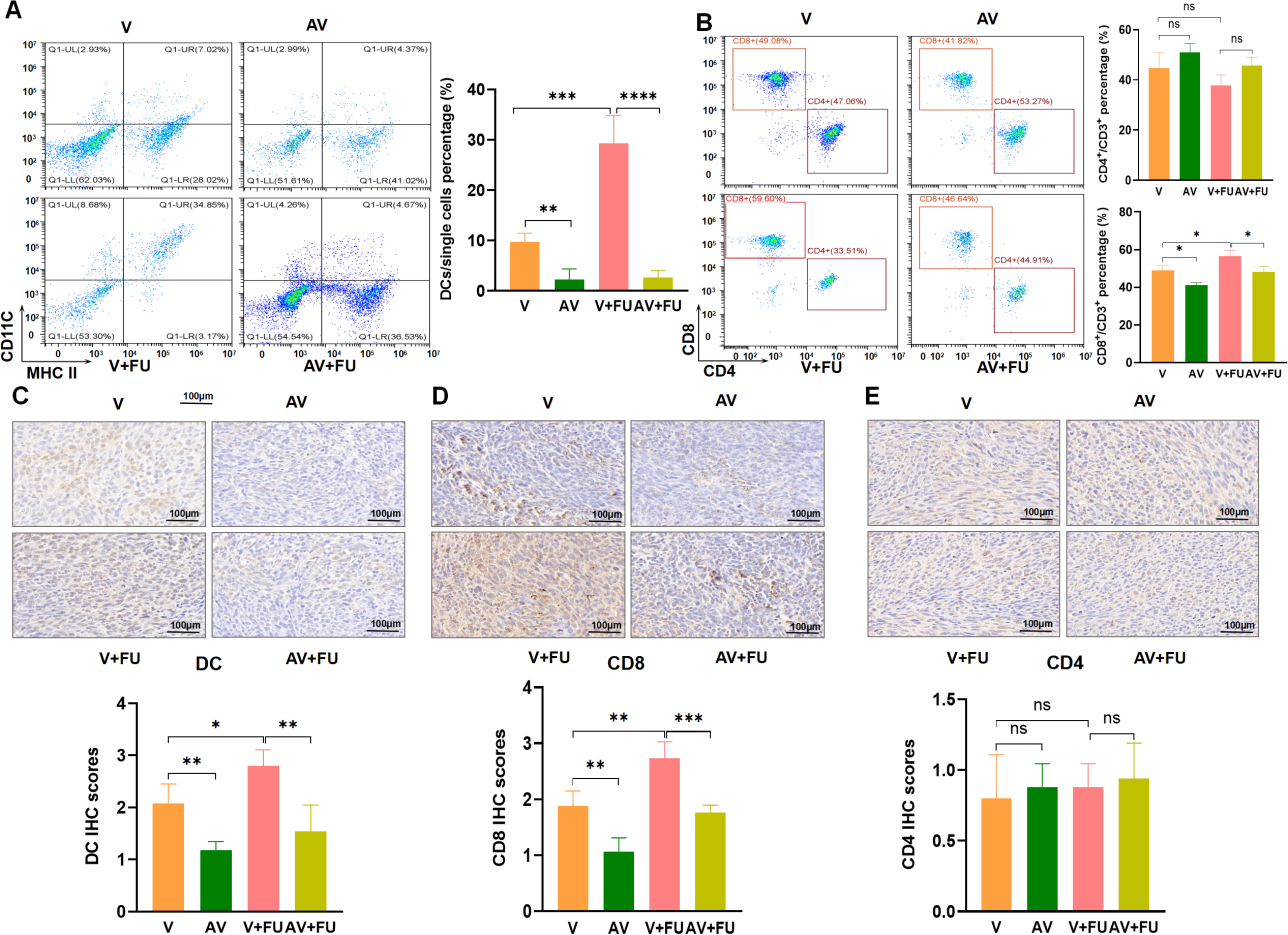
The dysbiosis of the gut microbiota induced by AV drugs reduces DCs and CD8+ T cells. **(A)** Flow cytometry analysis of DCs in different treatment groups (V, AV, V+FU, AV+FU). The bar chart illustrates the percentage of DCs among the total single cell population in each group. **(B)** Flow cytometry analysis of CD4+ and CD8+ T cells in different treatment groups. The bar chart shows the percentages of CD4+ and CD8+ T cells within the total CD3+ T cell population for each group. **(C)** Immunohistochemical staining and scoring of DCs in tumor tissues from different treatment groups. **(D)** Immunohistochemical staining and scoring of CD8+ T cells in tumor tissues from different treatment groups. **(E)** Immunohistochemical staining and scoring of CD4+ T cells in tumor tissues from different treatment groups. n=3. *, P < 0.05; ***, P < 0.001; ****, P < 0.0001.

To examine the impact of AV drug-induced gut microbiota dysbiosis on immune cells, we conducted flow cytometry to assess the cell number proportions of immune cells in peripheral blood and immunohistochemistry to evaluate immune cell infiltration in tumor tissues, based on the experimental design in **Figure 2A**. The results revealed that the number of DCs was significantly greater in the V+FU group than in the AV group or the AV+FU group (**Figure 6A**). The cell number proportion of CD8+ T cells in the V+FU group was also significantly higher than in the AV+FU group (**Figure 6B**). The cell number proportion of CD4+ T cells in the V group was lower compared to the AV group, and the cell number proportion of CD4+ T cells in the V+FU group was lower than in the AV+FU group, although these differences were not statistically significant (**Figure 6B**). Immunohistochemical staining of tumor tissues (**Figures 6C, D**) revealed greater numbers of DCs and CD8+ T cells in the V and V+FU groups than in the AV and AV+FU groups. There was no significant difference in the number of CD4+ T cells in any of the treatment groups (**Figure 6E**). These results suggest that AV treatment-induced gut microbiota dysbiosis reduces the numbers of DCs and CD8+ T cells in both peripheral blood (**Figures 6A, B**) and tumor tissues (**Figures 6C, D**), potentially impairing the immune response and the efficacy of 5-FU chemotherapy for treating CRC.

### 3.6 FMT restores the gut microbiota and increases DCs and CD8+ T cells

**Figure 7:**
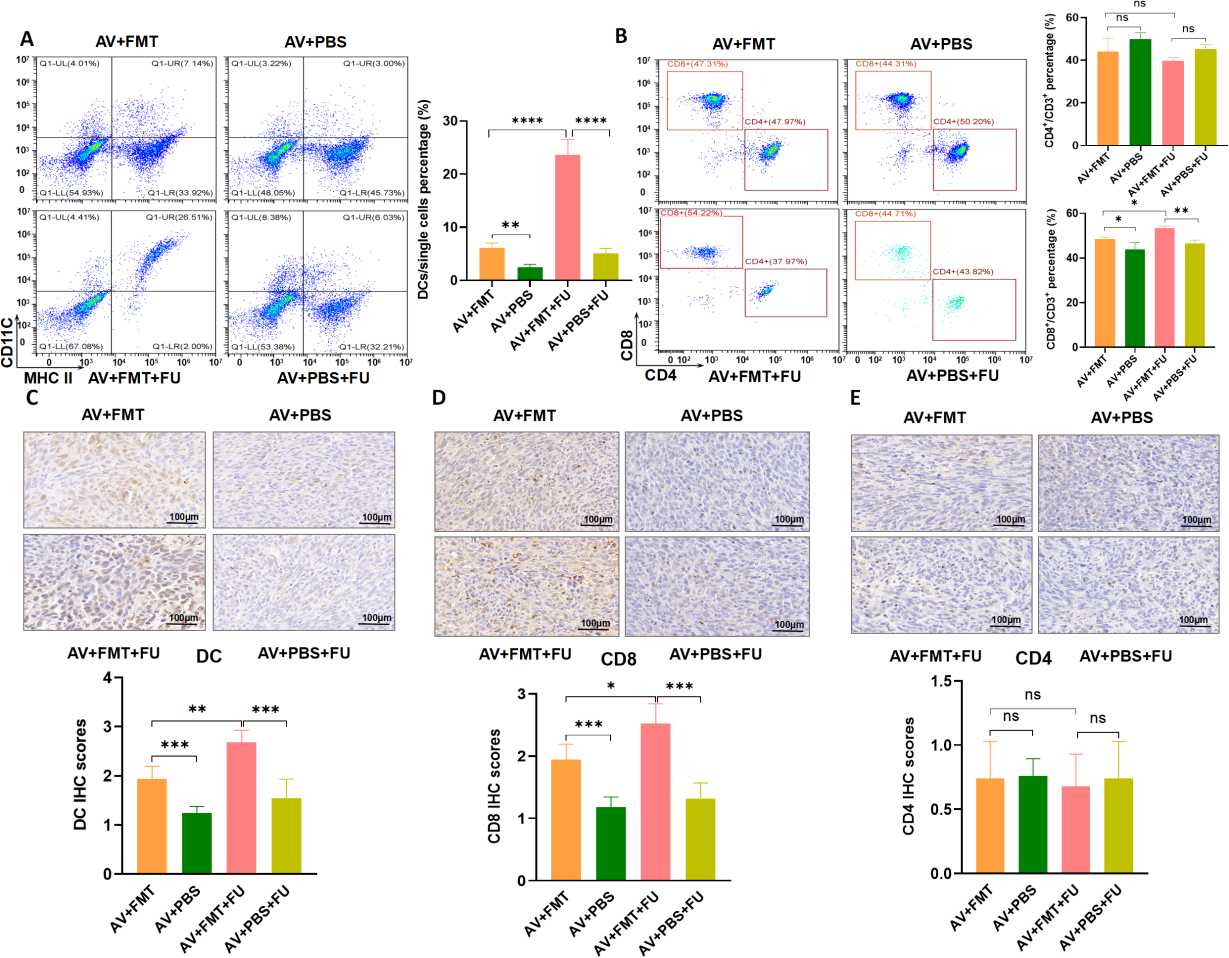
Fecal microbiota transplantation (FMT) restores the gut microbiota and increases DCs and CD8+ T cells. **(A)** Flow cytometry analysis of DCs in different treatment groups (AV+FMT, AV+PBS, AV+FMT+FU, AV+PBS+FU). The bar chart illustrates the percentage of DCs among the total single cell population in each group. **(B)** Flow cytometry analysis of CD4+ and CD8+ T cells in different treatment groups. The bar chart shows the percentages of CD4+ and CD8+ T cells within the total CD3+ T cell population for each group. **(C)** Immunohistochemical staining and scoring of DCs in tumor tissues from different treatment groups. **(D)** Immunohistochemical staining and scoring of CD8+ T cells in tumor tissues from different treatment groups. **(E)** Immunohistochemical staining and scoring of CD4+ T cells in tumor tissues from different treatment groups. n=3. *, P < 0.05; **, P < 0.01; ****, P < 0.0001.

To explore the effects of FMT on gut microbiota restoration and immune cell populations, we performed flow cytometry to assess the cell number proportions of immune cells in peripheral blood and immunohistochemistry to evaluate immune cell infiltration in tumor tissues, based on the experimental design in **Figure 3A**. Flow cytometry analysis revealed that the number of DCs was significantly greater in the AV+FMT+FU group than in the AV+PBS group and the AV+PBS+FU group (**Figure 7A**), indicating that FMT increases the number of DCs in the presence of 5-FU. The cell number proportion of CD8+ T cells in the AV+FMT+FU group was also significantly greater than that in the AV+PBS+FU group. The cell number proportion of CD4+ T cells in the V group was lower than in the AV group, and the cell number proportion of CD4+ T cells in the V+FU group was lower than in the AV+FU group, although these differences were not statistically significant (**Figure 7B**). Immunohistochemical staining of tumor tissues revealed that more DCs and CD8+ T cells were present in the AV+FMT group than in the AV+PBS group (**Figures 7C, D**). Additionally, the AV+FMT+FU group had more DCs and CD8+ T cells than did the AV+PBS+FU group. There was no significant difference in CD4+ T cell infiltration in all treatment groups (**Figure 7E**). These results suggest that FMT helps restore the gut microbiota and increases the numbers of DCs and CD8+ T cells in both peripheral blood (**Figures 7A, B**) and tumor tissues (**Figures 7C, D**), potentially improving the immune response and the efficacy of 5-FU chemotherapy for treating CRC.

### 3.7 Modulation of the TLR3-IRF3-IFN-β pathway by gut virome influences the efficacy of 5-FU chemotherapy in CRC

**Figure 8:**
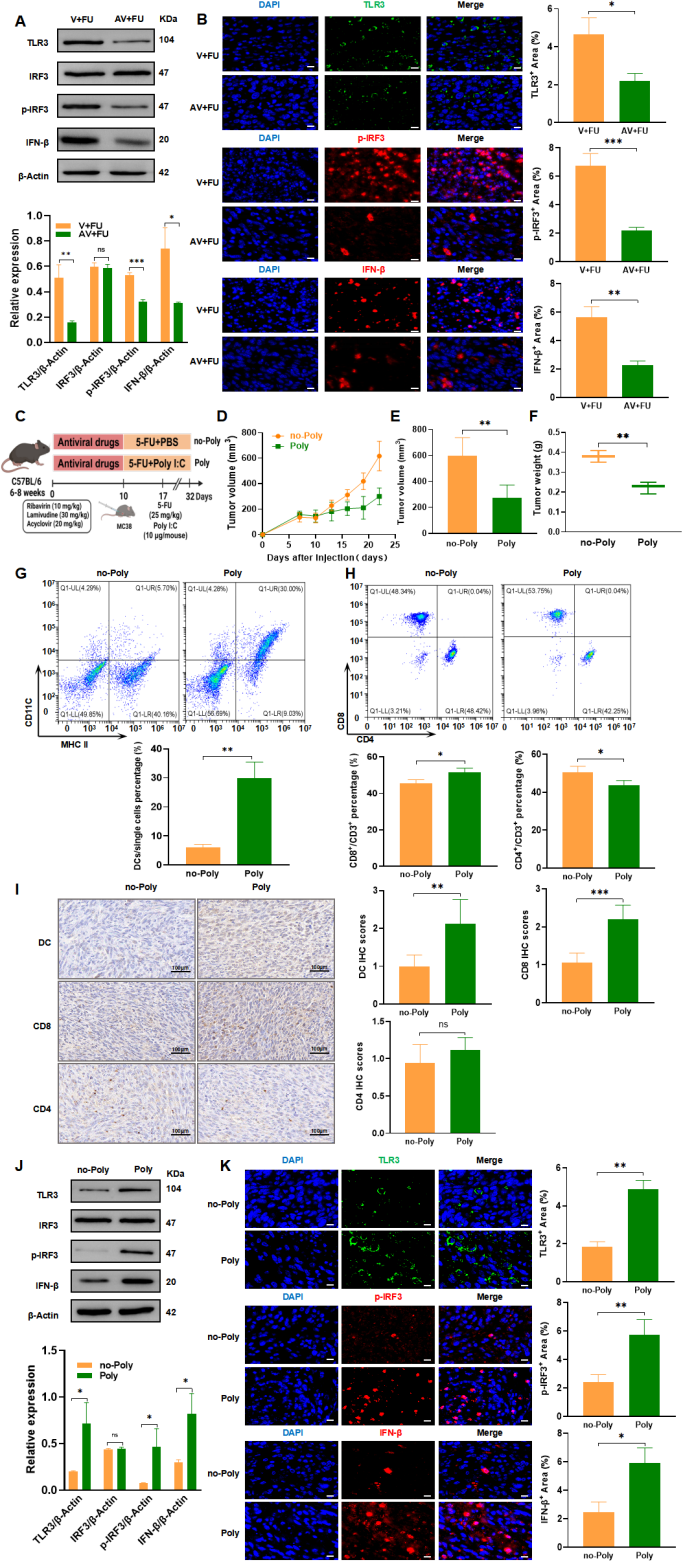
TLR3-IRF3-IFN-β pathway modulation by gut virome influences 5-FU efficacy in CRC treatment. **(A)** Western blot analysis of TLR3, IRF3, phosphorylated IRF3 (p-IRF3), and IFN-β expression in tumor tissues from the V+FU and AV+FU groups. β-Actin is used as a loading control. Quantification of band intensity is shown in the bar graph. **(B)** Immunofluorescence staining of TLR3, p-IRF3, and IFN-β in tumor sections from the V+FU and AV+FU groups. DAPI (blue) marks nuclei, while green and red fluorescence indicates TLR3, p-IRF3, and IFN-β expression, respectively. Quantitative analysis of fluorescence intensity is shown on the right. Scale bar=50 µm. **(C)** Schematic of experimental design: C57BL/6 mice were treated with antiviral drugs followed by 5-FU chemotherapy and the TLR3 agonist Poly(I:C). **(D)** Tumor volume measurements over time in Poly and no-Poly groups. **(E)** Comparison of final tumor volume on day 22 between Poly and no-Poly groups. **(F)** Comparison of tumor weights at the end of the experiment between Poly and no-Poly groups. **(G)** Flow cytometry analysis of DCs in different treatment groups (Poly and no-Poly). The bar chart illustrates the percentage of DCs among the total single cell population in each group. **(H)** Flow cytometry analysis of CD4+ and CD8+ T cells in different treatment groups (Poly and no-Poly). The bar chart shows the percentages of CD4+ and CD8+ T cells within the total CD3+ T cell population for each group. **(I)** Immunohistochemical staining of DCs, CD8+, and CD4+ T cells in tumor tissues from Poly and no-Poly groups. Scale bars=100 µm. Quantification of immunohistochemical scores for each cell type is shown to the right. **(J)** Western blot analysis of TLR3, IRF3, p-IRF3, and IFN-β expression in tumor tissues from Poly and no-Poly groups. β-Actin serves as the loading control. Quantification of protein expression levels is displayed in the bar graph. **(K)** Immunofluorescence staining of TLR3, p-IRF3, and IFN-β in tumor sections from Poly and no-Poly groups. DAPI (blue) highlights nuclei, while TLR3 (green), p-IRF3 (red), and IFN-β (red) are visualized in fluorescence channels. Quantitative analysis of each protein’s expression area is shown to the right. Scale bar=50 µm. n=3. *, P < 0.05; **, P < 0.01; ***, P < 0.001.

The TLR3-IRF3-IFN-β pathway is known to play a key role in detecting viral RNA and inducing antiviral immune responses, including type I interferon production, which is critical for enhancing immune cell functions like dendritic cell maturation and CD8+ T cell activation (16, 17). Given that the gut virome, composed of various viruses residing in the gut, can interact with host immune pathways like TLR3, there is potential for these viral communities to influence cancer immune responses and treatment efficacy. To explore whether modulation of the TLR3 pathway by the gut virome could impact CRC treatment, we investigated how antiviral-induced virome depletion and TLR3 activation through the agonist Poly(I:C) affect immune cell recruitment and the efficacy of 5-FU chemotherapy. Western blot analysis (**Figure 8A**) revealed that in the AV+FU group, expression of TLR3, p-IRF3, and IFN-β was reduced compared to the V+FU group, suggesting that antiviral treatment diminished TLR3 pathway activation. This finding was supported by immunofluorescence staining, which showed lower fluorescence intensities of TLR3, p-IRF3, and IFN-β in the AV+FU group compared to the V+FU group (**Figure 8B**), indicating that the presence of gut viruses may influence TLR3 activation levels.

We further explored whether TLR3 activation could restore immune responses and enhance the efficacy of 5-FU by treating mice with the TLR3 agonist Poly(I:C) in combination with 5-FU (experimental design shown in **Figure 8C**). Tumor measurements over time showed that Poly(I:C) treatment significantly inhibited tumor growth in the Poly group compared to the no-Poly group (**Figure 8D**), with both final tumor volumes and tumor weights lower in the Poly group (**Figures 8E, F**), indicating enhanced antitumor efficacy. Flow cytometry analysis of peripheral blood revealed that DC percentages were higher in the Poly group than in the no-Poly group, indicating increased recruitment of DCs following TLR3 activation (**Figure 8G**). Additionally, CD8+ T cell levels were elevated in the Poly group, while CD4+ T cell levels showed a downward trend in the Poly group compared to the no-Poly group, though this difference was not statistically significant (**Figure 8H**). Immunohistochemical staining of tumor tissues further confirmed increased infiltration of DCs and CD8+ T cells in the Poly group compared to the no-Poly group (**Figure 8I**). CD4+ T cell infiltration also showed a slight decrease in the Poly group, which aligned with the peripheral blood trends, but the difference was not statistically significant. Further validation through Western blot and immunofluorescence analyses demonstrated that Poly(I:C) treatment increased expression of TLR3, p-IRF3, and IFN-β in tumor tissues in the Poly group compared to the no-Poly group, confirming successful activation of the TLR3-IRF3-IFN-β pathway (**Figures 8J, K**). These findings collectively suggest that gut virome depletion reduces TLR3 pathway activation, subsequently limiting immune cell recruitment and potentially impairing 5-FU efficacy. Conversely, activating TLR3 with Poly(I:C) restores immune cell functions, enhancing the antitumor effects of 5-FU, thereby underscoring the potential role of gut virome interactions in optimizing CRC chemotherapy outcomes.

## 4 Discussion

CRC is one of the most prevalent malignancies worldwide, and chemotherapy remains an important treatment option, especially for patients with advanced-stage disease. However, responses to chemotherapy vary significantly among patients, with only 15–27% achieving complete remission, while 20–40% show minimal or no response to treatment (18). Therefore, there is an urgent need to better understand the factors affecting chemotherapy efficacy and to develop strategies to improve treatment outcomes for CRC patients. Recent studies have shown that the gut microbiome plays a crucial role in modulating the efficacy of chemotherapeutic drugs. The impact of antibiotics on gut bacteria and subsequent chemotherapy response has received considerable attention (8), but the role of the gut virome remains unclear. Therefore, in this study, we aimed to investigate how AV drug-induced gut virome dysbiosis affects the efficacy of 5-FU for treating CRC and to explore the underlying mechanisms involved.

Previous studies have focused mainly on the bacterial components of the gut microbiota, with limited attention given to the role of the gut virome. To investigate the relationship between the gut virome and chemotherapy efficacy in CRC patients, we collected fecal samples from 10 CRC patients before and after chemotherapy for metagenomic analysis. Based on the RECIST 1.1 tumor assessment criteria, these patients were divided into responders (4 patients with stable disease, 1 patient with partial response) and nonresponders (5 patients with disease progression). Our analysis revealed that the prechemotherapy responder (P-R) and nonresponder (P-NR) groups clustered separately from the postchemotherapy responder (Po-R) and nonresponder (Po-NR) groups, indicating that the gut virome structures differed between patients with different characteristics. However, the similarity of the viral communities between the P-R and Po-R groups suggests that responder viromes remained relatively stable despite chemotherapy, which may be one reason for their positive treatment response. The ACE index revealed that the viral diversity of nonresponders before and after chemotherapy was greater than that of responders, but the difference was not statistically significant. This observation suggests that a more diverse gut virome may be associated with a poor chemotherapy response. Analysis of the viral compositions of the different groups revealed that the gut viromes of the prechemotherapy nonresponder (P-NR) group were highly diverse, and genera such as *Punavirus*, *Delmidovirus*, and *Cohcovirus* were predominant. After chemotherapy, the relative abundances of *Afonbuvirus* and *Brigitvirus* increased in the postchemotherapy nonresponder (Po-NR) group, whereas the abundance of *Punavirus* decreased. These changes suggest that chemotherapy selectively promotes the expansion of certain viral genera while inhibiting others, which may contribute to poor chemotherapy outcomes. In contrast, the gut viromes of the prechemotherapy responder (P-R) group were dominated by *Afonbuvirus* and *Brigitvirus*, with lower abundances of *Punavirus* and *Delmidovirus*. In the postchemotherapy responder (Po-R) group, the abundances of *Brigitvirus* and *Ardovirus* increased, but the overall viral community structure remained relatively stable. This stable virome compositions of the responders may indicate that certain viral genera, such as *Brightvirus* and *Ardovirus*, play a protective role or support a gut environment favorable for the chemotherapeutic response. Further analysis of significantly differentially abundant viral taxa between pre- and postchemotherapy nonresponders and responders revealed key viral species whose abundances were associated with treatment outcomes. Before chemotherapy, *Enterococcus phage* was significantly enriched in the P-R group, suggesting that this phage may play a role in enhancing the chemotherapy response. In contrast, the P-NR group was significantly enriched in *Peduovirus pro483*, *Wadgaonvirus wv5004651*, *Gegavirus ST15OXA48phi141*, and *Xuanwuvirus xv520873*, indicating that these viruses may be associated with poor chemotherapy efficacy. After chemotherapy, *Cohcovirus splanchnicus*, *Cohcovirus hiberniae*, and *Jouyvirus ev207* were significantly enriched in the Po-R group, whereas *Stockinghalvirus FSLSP004* and *Lafunavirus LF1* were significantly enriched in the Po-NR group. These postchemotherapy changes suggest that certain viral species may support a continued positive response in responders or contribute to persistent poor treatment efficacy in nonresponders. The results of this study highlight the potential significance of the gut virome for influencing chemotherapy outcomes in CRC patients. The differences in the enrichment of specific viral taxa between responders and nonresponders suggest that the gut virome could serve as a biomarker for predicting chemotherapy efficacy. Identifying and understanding the roles of these viral taxa may help develop microbiome-based targeted therapies that enhance chemotherapy outcomes by modulating the gut virome. Additionally, the stability of the virome in responders despite chemotherapy treatment suggests that maintaining or restoring a specific viral community structure is critical for achieving better treatment outcomes. These findings demonstrate the possibility of using FMT to manipulate the gut virome to support the response to chemotherapy.

To further investigate the impact of gut viruses on 5-FU treatment for CRC, we employed an AV cocktail therapy (ribavirin, lamivudine, and acyclovir) to eliminate gut viruses in mice. After establishing a subcutaneous xenograft model with MC38 mouse colon cancer cells, we treated the mice with 5-FU and observed tumor growth. Our results showed that AV cocktail therapy significantly reduced the abundance of gut DNA and RNA viruses in mice. This is consistent with the findings of Yang et al. (13). Additionally, mice treated with AV drugs had larger tumor volumes and shorter survival times, indicating that AV drugs promote colorectal tumor growth. In mice treated with 5-FU, colorectal tumor growth was inhibited, and survival time was extended; however, in the AV-treated group, the antitumor effect of 5-FU was reduced. The potential antagonistic interaction between AV treatment and chemotherapy may be mediated by AV drug-induced gut microbiota dysbiosis. Previous studies have reported similar phenomena in animal models treated with antibiotics. Lu et al. reported that the combination of 5-FU and antibiotics significantly reduced the antitumor effect of 5-FU in a mouse CRC model (19). The potential mechanism may be that antibiotic-induced gut microbiota dysbiosis reduces the antitumor effect of 5-FU. In our study, we found that the reduction in viral abundance was consistent with increased tumor volume and shortened survival time, suggesting that AV drug-induced microbiota dysbiosis creates a gut environment favorable for tumor progression. Therefore, we hypothesize that the effects of AV drugs on colorectal tumor growth and 5-FU chemotherapy efficacy may be due to AV drug-induced gut microbiota dysbiosis. To verify this hypothesis, we performed FMT with healthy donor feces on recipient mice after AV drug treatment. We found that FMT restored the abundance of gut DNA and RNA viruses in mice treated with AV drugs. The tumor volumes of these mice were significantly reduced, and the survival time was significantly extended in FMT-treated mice compared with those not subjected to FMT. Additionally, colorectal tumor growth was inhibited, and survival time was extended in FMT-treated mice receiving 5-FU chemotherapy. These results suggest that restoring a healthy gut virome through FMT can enhance the therapeutic effect of 5-FU, possibly by reconstructing a gut environment that supports antitumor immunity. This finding is consistent with previous studies showing that FMT has the potential to restore the gut microbiota balance and improve CRC treatment outcomes. Previous studies have shown that transplanting healthy donor gut microbiota into patients to restore gut microbiota homeostasis may improve various gastrointestinal diseases, including irritable bowel syndrome, *Clostridioides difficile* infection, and CRC (20). John et al. reported that during CRC treatment, modulating the gut microbiota through FMT could restore chemotherapy-induced gut dysbiosis (21). These results suggest that the gut microbiota is closely related to colorectal tumor growth and chemotherapy efficacy.

To further study the effects of the gut microbiota on colorectal tumor growth and 5-FU chemotherapy efficacy, we performed metagenomic analysis on fecal samples from mice after gut virus depletion or FMT. Our results revealed distinct clustering of viral communities among the different treatment groups, indicating significant differences in the gut virome composition. The AV+FMT+FU and V+FU groups presented greater viral richness than the AV treatment groups did (AV+FMT+FU, AV+PBS+FU), although the differences were not statistically significant. Consistent with the findings of Li et al., the gut viromes of colorectal tumor-bearing mice exhibited significantly reduced α diversity and altered viral spectra (22), suggesting that AV treatment may reduce gut viral diversity, thereby affecting the gut environment and CRC progression. At the genus level, *Jundivirus communis*, *Goslarvirus goslar*, *Escherichia phage*, and *Cedarvirus Sf11* were the most abundant viral taxa across all groups. Notably, the viral community structures in the AV+FMT+FU group mice were similar to those in the V+FU group mice, suggesting that FMT may help restore a viral composition that is more conducive to inhibiting tumor growth. In contrast, AV treatment (AV+FU and AV+PBS+FU) led to increased relative abundances of *Jundivirus communis* and *Cedarvirus Sf11*, whereas the relative abundances of *Goslarvirus goslar* and *Escherichia phage* decreased. These changes suggest that AV treatment selectively promotes the development of certain viral taxa while inhibiting that of others, which may lead to a gut environment that supports CRC progression. LEfSe analysis further confirmed these findings by revealing significant differences in abundances of viral taxa between the V+FU and AV+FU groups. In the V+FU group, viruses such as *Salmonella phage*, *Felixounavirus*, and *Eponavirus* were enriched, indicating their dominance in the gut environment prior to AV treatment. In contrast, the AV+FU group was characterized by an enrichment of viral taxa such as *Kahnovirus*, *Petivirales*, and *Enterogokushovirus*, suggesting that AV treatment promotes the expansion of these viruses, which may negatively impact the efficacy of 5-FU treatment. In the AV+FMT+FU group, viral species such as *Peduovirus STYP1*, *Mahlunavirus rarus*, and *Jouyvirus ev207* were significantly enriched, indicating that FMT may create an environment that supports the proliferation of beneficial viral communities. Studies have shown that viruses, in addition to bacteria, play an important role in FMT; Zuo et al. reported that the therapeutic response to FMT in patients with *C. difficile* infection is associated with high colonization levels in the recipient of donor-derived tail-shaped bacteriophages (23). These findings suggest that FMT can modulate the gut virome composition, potentially restoring a more balanced and beneficial viral community that enhances the efficacy of 5-FU chemotherapy for treating CRC.

Our metagenomic analysis also revealed distinct clustering of bacterial and fungal communities among the different treatment groups, indicating compositional differences. However, α diversity analysis revealed no significant differences in bacterial or fungal diversity across the groups, suggesting that while the overall diversity remained stable, the specific compositions of these communities were altered by the treatments. Consistent with the findings of Yuan et al., AV drugs affected only a few specific bacterial taxa in the gut microbiome, with minimal effects on bacterial diversity. In the V+FU group, the gut bacterial community was predominantly composed of *Bacillota* and *Bacteroidota*, with lower proportions of other phyla, such as *Cyanobacteria* and *Actinomycetota*. The AV+FU group presented an increased abundance of *Bacteroidota* and *Cyanobacteria*, indicating that AV treatment alters the gut bacterial community in a manner that may disrupt the balance between beneficial and harmful bacteria. This disruption could contribute to the reduced efficacy of 5-FU treatment observed in the AV+FU group. In contrast, the AV+FMT+FU group exhibited a more distinct microbial profile, with greater differences in bacterial communities compared to both the AV+FU and V+FU groups. This group showed higher proportions of *Bacteroidota* and *Pseudomonadota*, suggesting that FMT had a significant impact on these bacterial populations. Such changes may contribute to creating a gut environment that is more favorable for enhancing the efficacy of 5-FU chemotherapy. Studies have revealed dysbiosis of the gut mycobiome in CRC patients, with an increased ratio of *Basidiomycota/Ascomycota* (24). In our study, the mycobiomes of all the groups were predominantly composed of *Ascomycota*, with minor contributions from *Basidiomycota* and *Chytridiomycota*. Notably, compared to the AV+PBS+FU group, the AV+FMT+FU group had higher proportions of *Basidiomycota* and *Chytridiomycota*, indicating that FMT supports the growth of these fungal communities. This shift may help improve 5-FU efficacy by promoting a gut environment that supports anti-tumor immunity and inhibits tumor progression. These results suggest that the gut microbiota is closely related to colorectal tumor growth and chemotherapy efficacy and that the role of gut viruses is particularly important compared with that of bacteria and fungi.

Cellular immunity plays a crucial role in tumor treatment, and immune cells exert antitumor immune responses through direct or indirect actions (25). Some studies have shown that chemotherapy-induced damage to living cells is a major determinant of T cell immunity (26). Other studies have reported a trend toward increased T cell proportions in tumors after chemotherapy, and traditional chemotherapeutic drugs may reshape the tumor immune microenvironment in some gastric cancer patients by recomposing T cell components and activating innate immune cells (27). Nakamura et al. reported that anthracycline drugs promote an increase in DC cells in tumor lesions through the chemokine CCL2 (28). Our results revealed that the number of DCs in the V+FU group was significantly greater than that in the AV group and AV+FU group. Similarly, the number of CD8+ T cells in the V+FU group was significantly greater than that in the AV+FU group. These results suggest that AV treatment induces gut microbiota dysbiosis, thereby reducing the numbers of DCs and CD8+ T cells in peripheral blood and tumor tissues. DCs are important antigen-presenting cells that play a key role in initiating and regulating immune responses, especially in activating CD8+ T cells (29). CD8+ T cells, also known as cytotoxic T cells, are key players in the directly killing of tumor cells (30). The reduced numbers of these cells suggest that AV treatment may impair the host’s ability to mount an effective immune response against CRC. Without a strong immune response, the ability of chemotherapy to target and destroy tumor cells is likely to be diminished. In contrast, we found that FMT restored the numbers of DCs and CD8+ T cells in the peripheral blood and tumor tissues. The numbers of these immune cells were significantly greater in the AV+FMT+FU group than in the AV+PBS and AV+PBS+FU groups. These findings suggest that FMT not only helps restore the gut microbiota but also enhances immune responses, thereby improving the antitumor effects of chemotherapy. The increase in DCs and CD8+ T cells following FMT indicates that restoring a balanced gut microbiota may reconstruct a more favorable immune environment. This is especially important in cancer treatment because a strong immune response is necessary for effectively eradicating tumors. We hypothesize that AV drug-induced gut microbiota dysbiosis may reduce the antitumor effect of 5-FU by suppressing the antitumor immune responses of DC and CD8+ T cells. However, the specific mechanisms involved remain to be further investigated.

The TLR3-IRF3-IFN-β signaling pathway is a critical component of innate immunity, recognized for its role in detecting viral infections and triggering antiviral responses. TLR3, a pattern recognition receptor, binds double-stranded RNA—a common feature of viral genomes—which initiates a signaling cascade through IRF3 (interferon regulatory factor 3) that ultimately leads to the production of IFN-β (16). IFN-β, a type I interferon, exerts several immunomodulatory effects, including enhancing dendritic cell maturation and promoting CD8+ T cell activation, both of which are crucial for mounting an effective antitumor immune response (17). Recently, studies have revealed that the gut virome can influence the host immune system by interacting with TLR3 signaling (13). This interaction may be essential in maintaining immune homeostasis and could influence cancer treatment outcomes. Thus, we hypothesized that modulating the gut virome could impact TLR3 pathway activation, potentially affecting immune cell dynamics and the efficacy of 5-FU chemotherapy in CRC. To test this hypothesis, we used antiviral treatment to deplete the gut virome in tumor-bearing mice and examined its impact on the TLR3-IRF3-IFN-β pathway activation and immune cell populations. We also explored whether activating TLR3 with the agonist Poly(I:C) could counteract the effects of virome depletion and improve the efficacy of 5-FU chemotherapy.

In this study, antiviral treatment aimed at depleting the gut virome reduced TLR3 pathway activation, as evidenced by decreased expression of TLR3, p-IRF3, and IFN-β in the AV+FU group. This downregulation in the TLR3-IRF3-IFN-β pathway coincided with lower levels of DC and CD8+ T cell populations, suggesting that the absence of viral components in the gut microbiome reduces the activation signals necessary for maintaining a robust antitumor immune response. Since DCs and CD8+ T cells are essential in detecting and eliminating tumor cells, the reduction in these cell populations likely compromised the effectiveness of 5-FU, as indicated by larger tumor volumes and weights in the AV+FU group. Conversely, activating the TLR3 pathway using the agonist Poly(I:C) restored TLR3 signaling and promoted the expression of IFN-β and downstream immune responses. The Poly+5-FU combination treatment led to increased DC and CD8+ T cell infiltration in tumor tissues, supporting the hypothesis that TLR3 activation can counterbalance the immunosuppressive effects of virome depletion. This observation suggests that TLR3 pathway activation facilitates the recruitment and function of immune cells within the tumor microenvironment, enhancing the overall efficacy of 5-FU chemotherapy. The role of TLR3 in boosting IFN-β levels is particularly relevant, as IFN-β not only aids in viral clearance but also supports an immune-permissive tumor environment by increasing antigen presentation and stimulating CD8+ T cell responses, which are essential for effective chemotherapy. The observed differences in CD4+ T cell dynamics between the treatment groups add further nuance to the interpretation of TLR3’s role in CRC. While CD4+ T cell levels exhibited a trend of reduction in the Poly group compared to the no-Poly group, this difference was not statistically significant. The slight decrease in CD4+ T cells suggests that TLR3 activation may selectively enhance cytotoxic CD8+ T cell responses. This selective recruitment of CD8+ T cells over CD4+ T cells may be an advantageous mechanism in CRC, where increased cytotoxic activity can directly limit tumor progression.

The findings of this study emphasize the importance of the gut virome as an immunomodulatory element, capable of enhancing chemotherapy efficacy through TLR3-mediated signaling. Gut viral communities, by interacting with host immune receptors like TLR3, may act as natural adjuvants, triggering innate immune responses that potentiate antitumor activity. Thus, the depletion of these viral components through antiviral treatments could inadvertently suppress beneficial immune pathways, underscoring the complexity of gut microbiota and virome interactions in cancer therapy. Targeting the gut virome or employing TLR3 agonists like Poly(I:C) could be strategic for enhancing immune responses, particularly in cases where patients have undergone antiviral treatments that may reduce gut microbial diversity and function. Future research could further investigate the precise viral components within the gut virome that are essential for TLR3 pathway activation, which may lead to the development of tailored microbial therapies or TLR3-based immunomodulatory strategies for CRC patients.

Although this study reveals the impact of AV drug-induced gut microbiota dysbiosis on the efficacy of chemotherapy for treatment of CRC, several limitations remain. First, the clinical study had a small sample size of 10 CRC patients, limiting the generalizability of the findings. A larger cohort would provide more robust data and help validate the observed associations between the abundance of specific viral taxa and chemotherapy outcomes. Second, this study focused primarily on changes in the gut virome and their impact on chemotherapy efficacy, with limited investigations into the combined effects of other microbiota, such as gut bacteria, fungi, and archaea. Since these microbiota may play important roles in host immunity and tumor progression, future research should aim to elucidate these complex interactions to better understand the mechanisms by which the gut microbiota influences CRC treatment outcomes.

## 5 Conclusion

This study reveals that AV drug-induced gut virome dysbiosis impairs the TLR3-IRF3-IFN-β pathway, reducing the numbers of immune cells, such as DCs and CD8+ T cells, which are crucial for an effective antitumor response in CRC. This impairment diminishes the efficacy of 5-FU chemotherapy, as shown by increased tumor growth and reduced survival in AV-treated mice. Conversely, activation of the TLR3 pathway through Poly(I:C) enhances immune function, restores DC and CD8+ T cell populations, and improves the antitumor effects of 5-FU, suggesting a potential strategy to counteract the negative impact of virome depletion on chemotherapy. These findings underscore the importance of maintaining gut virome integrity or activating TLR3 as adjunct strategies to enhance chemotherapy outcomes in CRC patients.

## Data Availability

The metagenomic sequencing data generated in this study have been deposited in the Sequence Read Archive (SRA) of the National Center for Biotechnology Information (NCBI) and can be accessed via the following URL: https://www.ncbi.nlm.nih.gov/sra/ PRJNA1195998; https://www.ncbi.nlm.nih.gov/sra/ PRJNA1196101.
